# A Promotion Role of MIR31 in the Process of Vocal Fold Wound Healing

**DOI:** 10.1155/2023/4672827

**Published:** 2023-08-08

**Authors:** Haizhou Wang, Wen Xu

**Affiliations:** ^1^Department of Otolaryngology-Head and Neck Surgery, Beijing Tongren Hospital, Capital Medical University, Beijing, China; ^2^Key Laboratory of Otolaryngology-Head and Neck Surgery, Ministry of Education of China, Beijing, China

## Abstract

The role of MIR31 in the wound healing process, specifically in vocal fold wound healing (VFWH), remains uncertain despite its potential to facilitate the process. In this study, we first constructed a literature-based pathway to examine both the positive and negative effects of MIR31 on wound healing. We then conducted animal experiments on 20 rats to investigate MIR31 expression at different time points (1, 4, and 8 weeks) after vocal fold injury. Co-expression analysis and pathway analysis were performed to explore the potential function of MIR31 in VFWH. The literature-based pathway suggested that MIR31 could both impede and promote the wound healing process by regulating 14 and 47 wound healing upstream regulators, respectively. However, the rat experiment indicated that MIR31 expression significantly increased after vocal fold injury (*p* < 5.65 × 10^−5^) but decreased in the late stage of VFWH compared with the early and middle stages (*p* < 5.40 × 10^−3^. Strong co-expression was observed between MIR31 and 17 VFWH-significant genes (Pearson correlation coefficient ∈ (0.63, 0.83)), primarily involved in collagen production. Overall, our findings suggest that MIR31 plays a critical role in VFWH, particularly in collagen synthesis and other biological processes, which warrant further investigation.

## 1. Introduction

The structure of the extracellular matrix (ECM) is essential for the functionality of the vocal fold to produce sounds [1]. Vocal fold injury could lead to abnormal massive hyperplasia of the fibrous tissue and ECM disorder in the lamina propria. Vocal fold injury caused fibrous scars that could bring persistent pararthria and irreversible changes [2–4]. So far, it is still a challenging clinical topic to treat vocal fold scars effectively.

Vocal fold wound healing (VFWH) could take from two weeks to several months [5, 6]. Multiple genes and proteins have been shown to play roles in the wound healing process [7]. For instance, Mothers Against Decapentaplegic Homolog 1 (SMAD1) has been suggested as a therapeutic molecular target in skin wound healing that plays an active role in wound repair and regenerative medicine [7, 8]. Spallotta et al.'s study showed that histone deacetylase 2 (HDAC2) inhibition could significantly enhance skin wound repair [8].

MIR31 is a short non-coding RNA that is involved in the post-transcriptional regulation of gene expression in multicellular organisms by affecting both the stability and translation of messenger RNA (mRNAs) [9, 10]. Multiple previous studies suggested that MIR31 plays a role in the pathology of skin wound healing, including the stimulation of wound contraction to enhance wound closure [11] and the promotion of skin wound healing by enhancing keratinocyte proliferation and migration [12]. Despite its potential to promote wound healing, MIR31 can also hinder the process by inhibiting wound healing promoters, such as Peroxisome Proliferator-Activated Receptor Gamma (PPARG), Interleukin-6 (IL6), and C-C Motif Chemokine Ligand 5 (CCL5) [13–15], and promoting wound healing inhibitors like MKI67, ACAN, and AHR [16–18]. Hence, further research is needed to fully understand the effects of MIR31 on wound healing. Currently, there is no research on whether MIR31 plays a role in VFWH, making it an area that requires further investigation.

In this study, a microarray was used to detect the expression changes of MIR31 in different stages of VFWH. In addition, a co-expression analysis was conducted between MIR31 and the genes that presented significant expression changes in the vocal fold after injury. Our results indicated that the expression of MIR31 was stimulated by the wound and was significantly related to the vocal fold's wound healing process. This study guarantees further study to explore the role of MIR31 and VFWH.

## 2. Methods

### 2.1. mRNA and miRNA Data Extraction

The animal experiments in this study were performed by the regulations of the Peking University Animal Care and Use Committee (LSC-ZhangY-1) and license permit: 1116012800123. Male Sprague–Dawley (SD) rats aged 14–16 weeks and weighing 400–450 g underwent unilateral or bilateral vocal fold injury using procedures described in an earlier study [19]. The vocal fold was injured by separating and removing the lamina propria from the thyroarytenoid muscle. First, 20 animals were randomly divided into four experimental groups (five animals in each group) based on time of sacrifice: uninjured control, and 1, 4, and 8 weeks after injury. Following sacrifice, the larynx was harvested, and the bilateral vocal folds were dissected under magnification. Each specimen was immediately snap-frozen in liquid nitrogen and stored at −80°C for subsequent study.

Total RNA, including both mRNA and micro RNA (miRNA), was extracted from vocal folds using TRIzol Reagent (Life Technologies, Carlsbad, CA, USA) and purified with an RNeasy mini kit (Qiagen, Valencia, CA, USA). Biotinylated complementary DNA (cDNA) was prepared according to the standard Affymetrix protocol from 250 ng of total RNA using an Ambion® WT Expression Kit. Following labeling, fragmented cDNA was hybridized for 16 hours at 45°C with the Clariom™ S Assay (rat, Affymetrix). GeneChips were washed and stained in the Affymetrix Fluidics Station 450. All arrays were scanned using an Affymetrix® GeneChip Command Console, which was installed in a GeneChip® Scanner 3000 7G.

The row data (.cel) were normalized using the TAC software (Transcriptome Analysis Console, Version 4.0.1) with the Robust Multichip Analysis (RMA) algorithm using Affymetrix default analysis settings and global scaling as the normalization method. Values presented are log2 RMA signal intensity. The microarray data discussed in this study are submitted to NCBI Gene Expression Omnibus with accession number GSE139383.

### 2.2. Screening of Differentially Expressed Genes

To analyze the mRNA and miRNA expression data, we initially utilized the Limma R package (version 3.36.5). Our goal was to examine the expression of MIR31 across various groups and identify the differentially expressed genes (DEGs) in the injured groups compared with the uninjured control group. To control the false discovery rate (FDR) for multiple tests, we employed the Benjamini–Hochberg procedure with a significance threshold of *q* = 0.05. The criteria for up- and downregulated genes were set as fold change >1.5 or <−1.5, with an FDR-corrected *p*-value <0.01. Subsequently, we employed one-way Analysis of Variance (ANOVA) to detect the expression changes of both MIR31 and other DEGs that exhibited significant expression variations in the three injured groups (*p* < 0.01). We further investigated the co-expression between MIR31 and the DEGs to explore potential associations between them.

For further analysis, we selected genes that met the following criteria: (1) they showed significance in each injured group compared with the control group (fold change >1.5 or <−1.5, and *p* < 0.01); (2) they also demonstrated significance when compared among the three injured groups (*p* < 0.01).

### 2.3. Functional Network and Pathway Analysis

A literature-based functional network analysis was carried out to investigate the genetic connection between MIR31 and wound healing. This analysis revealed molecules regulated by MIR31 that play a role in the wound healing process. The network analysis relied on Pathway Studio (http://www.pathwaystudio.com) for assistance. By comparing the downstream targets of MIR31 with the upstream regulators of wound healing, the genetic pathways driven by MIR31 were constructed. These pathways have the potential to either promote or inhibit the wound healing process. Additionally, a gene set enrichment analysis (GSEA) was conducted using Gene Ontology (GO) terms and Human Protein Atlas Expression Ontology (HPAEO). This analysis provided insights into the functional profile of MIR31 and its related DEGs. Pathways/GO items show significance were reported (FDR corrected *p* < 0.05 and overlap ≥2).

## 3. Results

### 3.1. Possible Negative Role of MIR31 on Wound Healing

**Figure 1:**
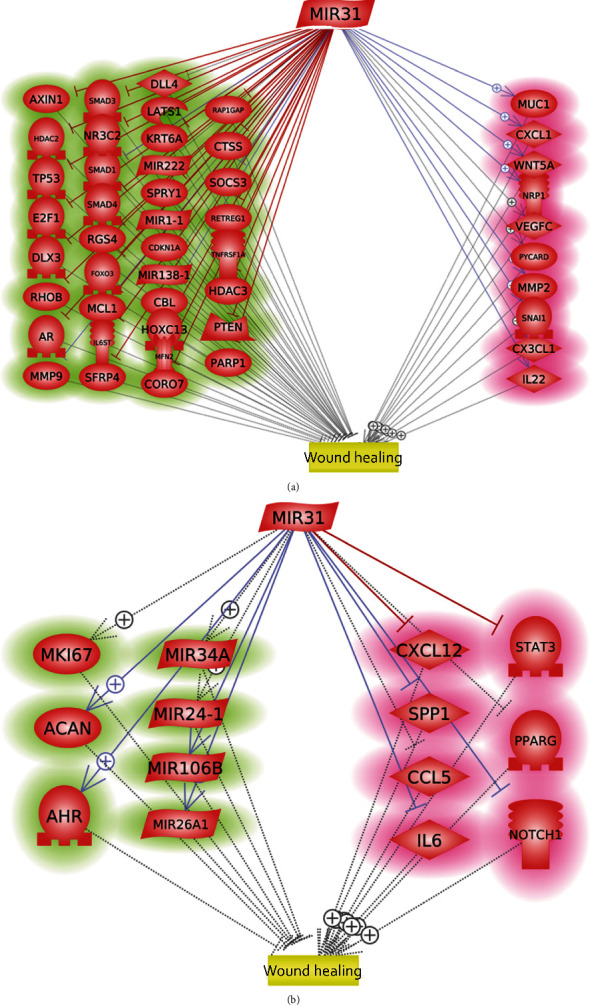
MIR31-driven molecules related to wound healing. (a) MIR31-driven molecules promote wound healing, including 37 wound healing inhibitors (highlighted in green); and 10 wound healing promoters (highlighted in red). (b) MIR31-driven molecules hinder the wound healing process, including the 7 wound healing inhibitors (highlighted in green) and 6 wound healing promoters (highlighted in red).

Literature-based pathway analysis revealed that MIR31 can stimulate wound healing by activating 10 wound healing promoters and inhibiting 37 wound healing inhibitors, as illustrated in Figure 1(a).

However, as shown in Figure 1(b), MIR31 may also hinder the wound healing process by inhibiting seven wound healing promoters, including CXCL12, SPP1, STAT3, NOTCH1, PPARG, IL6, and CCL5. MIR31 can also promote seven wound healing inhibitors, including MKI67, ACAN, AHR, MIR34A, MIR24-1, MIR106B, and MIR26A1. Therefore, further research is needed to fully understand the effects of MIR31 on wound healing, especially for VFWH.

### 3.2. MIR31 Expression in VFWH

**Figure 2:**
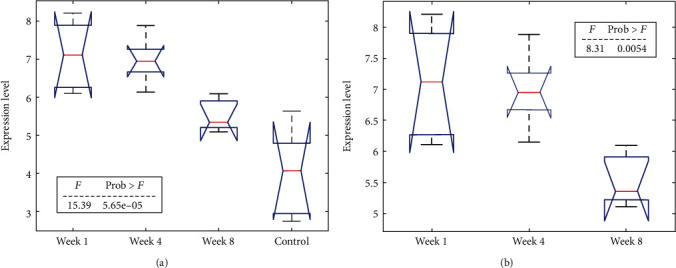
Comparison of expression levels of MIR31 in the four groups of rats. (a) The comparison results of four groups. (b) The comparison results of three injured groups.

When compared with uninjured vocal fold tissues (control group, *n* = 5), MIR31 demonstrated significantly increased expression in injured vocal fold tissues at different time points (1, 4, and 8 weeks; *n* = 5 in each experimental group) with *p* < 5.65 × 10^−5^, as shown in Figure 2(a). It was found that the expression of MIR31 also varied significantly among injured vocal fold tissues at different time points (*p* < 0.0054; see Figure 2(b)).

### 3.3. Expression of 15 Significant Genes

**Figure 3:**
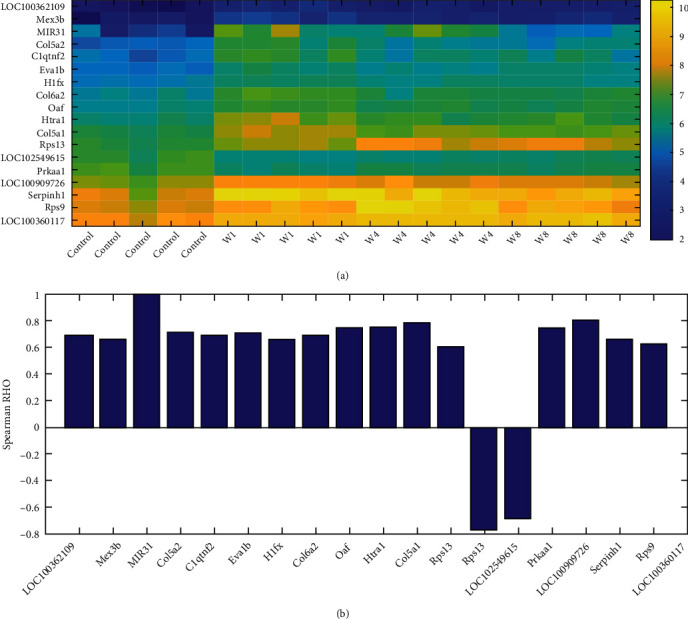
Expression and co-expression of 17 genes and MIR31 in the four groups of rats. (a) Heat map of expression levels. (b) Correlation between the 18 genes and MIR31.

Besides MIR31, our analysis also identified 17 other genes that present significance when compared among both four groups (*p* < 0.0099) and three injured groups (*p* < 0.0075), including *LOC100362109*, *Mex3b*, *Col5a2*, *C1qtnf2*, *Eva1b*, *H1fx*, *Col6a2*, *Oaf*, *Htra1*, *Col5a1*, *Rps13*, *LOC102549615*, *Prkaa1*, *LOC100909726*, *Serpinh1*, *Rps9*, and *LOC100360117*. The expression heat map of these genes and MIR31 was presented in Figure 3(a).

Interestingly, 15 out of the 17 genes presented a strong positive correlation with MIR31 in terms of expression variation among the four groups (ROH ∈ (0.63, 0.83)), and two showed a robust negative correlation with MIR31 (ROH = −0.78 and −0.70, respectively), as shown in Figure 3(b). These results suggested that MIR31 may regulate or co-function with these genes to influence the wound healing process of the vocal fold.

### 3.4. GSEA Results

**Figure 4:**
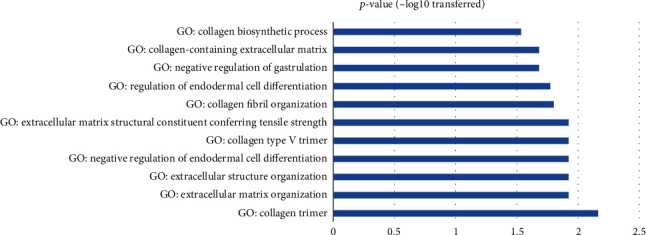
GSEA results of 17 genes and MIR31.

Enrichment analysis was conducted using GSEA to compare the 17 genes (refer to Figure 3(a)) and MIR31 with the GO terms and HPAEO terms. The enrichment analysis utilized these 18 items as input. Results showed that these 17 genes were mainly involved in collagen-related GO terms, as shown in Figure 4. No HPAEO gene group was identified. Supplementary Material 1 presents comprehensive information on these pathways, encompassing the GO ID, the number of entities, the overlapping genes, as well as the *p*-values before and after FDR.

## 4. Discussion

Previous studies have suggested that MIR31 may facilitate skin wound healing by promoting the proliferation and migration of keratinocytes [11, 12]. However, MIR31 may also hinder the wound healing process by inhibiting seven wound healing promoters, namely CXCL12, SPP1, STAT3, NOTCH1, PPARG, IL6, and CCL5 [13–15]. Moreover, MIR31 can promote seven wound healing inhibitors, including MKI67, ACAN, AHR, MIR34A, MIR24-1, MIR106B, and MIR26A1 [16–18]. Therefore, further investigations are required to comprehensively comprehend the impact of MIR31 on wound healing, particularly in the context of VFWH. Results from this study showed that MIR31 was stimulated by vocal fold wounds to demonstrate significantly high expression at the initial stage of vocal fold healing, which decreased in the late stage of the wound healing process. Moreover, MIR31 demonstrated a strong correlation with 17 genes showing significant expression variation during VFWH, which were mainly involved in collagen synthesis. Our results suggest that MIR31 is closely related to VFWH.

Expression analysis showed that MIR31 expression was significantly increased at the initial stage of VFWH (week 1), as shown in Figure 2(a). Upregulated MIR31 has been reported to alleviate inflammation in colon injury [20] and cardiac injury [21]. At the early stage of wound healing, inflammation is the initial response to cellular injuries and is the key process in wound healing [22]. MIR31 has also been shown to stimulate wound contraction and thus enhance wound closure [11]. Therefore, our results indicate that increased MIR31 expression could help at the early stage of the VFWH.

The up-regulated expression of MIR31 lasted to week 3 without an obvious decrease, as shown in Figure 2(b). MIR31 has been shown to promote skin wound healing by enhancing keratinocyte proliferation and migration, which may happen through suppressing its direct target gene, epithelial membrane protein 1, during wound healing [12]. MIR31 could also suppress the inhibitors of wound healing, including SMAD1 [23] and HDAC2 [24]. Moreover, up-regulation of MIR31 could lead to elevated cellular adenosine triphosphate (ATP) that is required for wound closure [25, 26]. Therefore, lasted overexpression of MIR31 could exert continued aid to the VFWH.

We also noticed that MIR31 expression significantly dropped 8 weeks after the vocal fold wound (*p* < 0.0054), which may be corresponding to the late stage of the wound healing process (Figure 2(b)). However, this may also reflect balanced factors that influenced VFWH. Figure 1(b) illustrates that MIR31 can impede the wound healing process by blocking various agents that promote wound healing, whereas encouraging the presence of certain factors that inhibit it. For instance, PPARG, a nuclear hormone receptor, plays a crucial role in wound healing by regulating inflammation, tissue remodeling, and cell signaling [13]. Activation of PPARG promotes wound healing by reducing oxidative stress, suppressing inflammation, and increasing the expression of wound-healing genes [27]. Additionally, PPARG deficiency or inhibition leads to impaired wound healing due to delays in apoptotic cell clearance, dysfunctional adipocytes, and fibrosis inhibition [28]. Consequently, the decline in MIR31 expression during later stages could facilitate the activation of these corresponding regulators that aid in wound healing. Nevertheless, additional research is required to fully understand the mechanism of MIR31 expression regulation during different stages of the VFWH process.

Co-expression analysis showed that MIR31 was strongly related to 17 genes that presented significant expression changes during VFWH (Figure 3). These genes were mainly involved in collagen biosynthetic and trimming, as shown in the GSEA results presented in Figure 4. Collagen has been shown to increase keratinocyte proliferation, positively acting on cell entry in the mitotic phase [29]. Thus, collagen synthesis and deposition into the wound are essential during wound healing [30]. Our results suggested that MIR31 may co-function with the 17 collagen production regulators to play a role in the VFWH process. In addition, our study also showed that MIR31 was related to multiple biological processes that were linked to wound healing, including fibrosis and keloid [31, 32].

Furthermore, investigation is required to address the limitations of this study in the future. First, it is crucial to validate the results obtained from animal studies by incorporating human data. Additionally, the pathway depicted in Figure 1 relies heavily on previous publications and should be subjected to human experimentation for further confirmation.

## 5. Conclusion

This study supports the promotion role of MIR31 in the process of VFWH, which may be through the regulation of multiple biological processes, including keratinocyte proliferation, collagen production, ATP generation, and inflammation.

## Data Availability

Upon contacting the corresponding author, all data generated or analyzed during this study can be accessed.

## Supplementary Materials

Supplementary Materials Supplementary Material 1 presents comprehensive information on these pathways, encompassing the GO ID, the number of entities, the overlapping genes, as well as the *p*-values before and after FDR.
