# Supplementary material for: A Promotion Role of MIR31 in the Process of Vocal Fold Wound Healing
Source: PPAR Res. 2023 Aug 8;2023:4672827. doi: 10.1155/2023/4672827 (PMC10427237; doi:10.1155/2023/4672827)
Supplement: Supplementary Materials — Supplementary Material 1 presents comprehensive information on these pathways, encompassing the GO ID, the number of entities, the overlapping genes, as well as the p-values before and after FDR. [file 4672827.f1.pdf]

Name	GO ID
GO: collagen trimer	0005581;
GO: extracellular matrix organization	0030198;
GO: extracellular structure organization	0043062;
GO: negative regulation of endodermal cell differentiation	1903225;
GO: collagen type V trimer	0005588;
GO: extracellular matrix structural constituent conferring tensile strength	0030020;
GO: collagen fibril organization	0030199;
GO: regulation of endodermal cell differentiation	1903224;
GO: negative regulation of gastrulation	2000542;
GO: collagen-containing extracellular matrix	0062023;
GO: collagen biosynthetic process	0032964;

# of Entities	Overlap	Overlapping Genes	p-value	p-value_beforeFDR
100	4	C1QTNF2;COL5A1;COL5A2;COL6A2	0.0068	1.696E-07
411	5	HTRA1;SERPINH1;COL5A1;COL5A2;COL6A2	0.01191	9.479E-07
412	5	HTRA1;SERPINH1;COL5A1;COL5A2;COL6A2	0.01191	9.593E-07
4	2	COL5A1;COL5A2	0.01191	1.328E-06
4	2	COL5A1;COL5A2	0.01191	1.506E-06
43	3	COL5A1;COL5A2;COL6A2	0.01191	1.781E-06
58	3	SERPINH1;COL5A1;COL5A2	0.01579	2.756E-06
6	2	COL5A1;COL5A2	0.01663	3.318E-06
7	2	COL5A1;COL5A2	0.02057	4.644E-06
538	5	HTRA1;SERPINH1;COL5A1;COL5A2;COL6A2	0.02057	5.128E-06
9	2	SERPINH1;COL5A1	0.02901	7.956E-06
